# Let’s Talk About Voices: randomised controlled crossover study of a resource to support mental health workers in supporting voice-hearers

**DOI:** 10.1192/bjo.2025.10071

**Published:** 2025-07-07

**Authors:** Anne Honey, Justin Newton Scanlan, Lyndal Sherwin, Haylee Zink, Karen Wells, Glenda Jessup, Nicola Hancock

**Affiliations:** Centre for Disability Research and Policy, Faculty of Medicine and Health, The University of Sydney, Australia; Northern Sydney Local Health District, NSW Health, Sydney, Australia

**Keywords:** Psychosis, schizophrenia, psychosocial intervention, hearing voices approach

## Abstract

**Background:**

Easily accessible, impactful, evidence-based resources are needed to assist mental health workers to best support voice-hearers in managing and living well with voices. Let’s Talk About Voices (LTAV) is an innovative suite of resources designed for mental health workers to use in supporting voice-hearers.

**Aim:**

This study aimed to assess the impact of LTAV on mental health workers’ self-reported capacity to work with voice-hearers.

**Method:**

A randomised, controlled crossover design was used, with assessment at three time points. The assessment measure was co-developed by researchers, clinicians, peer workers and voice-hearers based on the aims of LTAV and the Theory of Planned Behaviour. Participants were randomised into two groups. The immediate group received access to LTAV following the first assessment; the delayed group received access following the second assessment. In total, 256 mental health workers commenced the study, with 120 completing all assessments.

**Results:**

Between-group comparisons for change between times 1 and 2 found a significant difference with a large effect size (*F* = 40.2, *P* < 0.001, *η_p_*^2^ = 0.19). Significance remained on intention-to-treat analysis (*F* = 22.9, *P* < 0.001, *η_p_*^2^ = 0.08). Pairwise repeated-measures comparisons found a significant increase in scores for both groups following access to LTAV, which was sustained at follow-up. Fourteen of 24 individual items showed significant change. Changes were consistent across professions, work settings and experience working with voice-hearers, but those with less confidence in working with voice-hearers on intake showed significantly stronger improvements.

**Conclusions:**

This research indicates that LTAV has the potential to substantially improve mental health workers’ attitudes and confidence in supporting voice-hearers.

Voice-hearing is experienced by around 75% of people diagnosed with schizophrenia.^1^ The impact of antipsychotic medication is variable, and many people continue to experience voice-hearing.^2,3^ It is increasingly recognised that a non-medical, recovery-oriented approach is needed to support voice-hearers. This is in line with increasing expectations and requirements from governments and mental health policies for mental health services to adopt a recovery-oriented approach.^4,5^ In this context, ‘recovery’ does not refer to erasing symptoms but to living a meaningful, socially connected and personally valued life.^6^ Adopting a recovery-oriented approach to voice-hearing means supporting voice-hearers to flourish and do the things that they want to do, even in the presence of ongoing voices.

The hearing voices approach^7^ challenges common biologically oriented conceptualisations of voices as meaningless symptoms of illness to be suppressed or ignored. Rather, voice-hearing is seen as a meaningful part of human experience and a response to people’s histories and circumstances, including trauma.^8^ Furthermore, the impact of voices on a person’s distress in daily life is largely determined by their interpretation of those voices.^9^ By exploring, understanding and changing their relationships with their voices, voice-hearers can learn to accept, cope with and successfully manage their voices.^10^ A systematic review and synthesis of qualitative research about recovery processes in voice-hearing found that making sense of voice-hearing experiences, accepting voices and changing one’s beliefs about and relationships with voices facilitated recovery.^11^ The reviewers identified that searching for meaning in voices tended to result in less continued distress than blocking out or fighting against voices, which was often associated with seeing them as meaningless symptoms.

The hearing voices approach is being used increasingly across the world,^12^ predominantly in voice-hearing groups, which have been found to have benefits including improved coping skills, developing a better understanding of and a different relationship with voices, reducing stress and improving self-esteem.^13^ Participants also reported that these groups provided a kind of support unavailable to them elsewhere.^13^

Nevertheless, not all voice-hearers have access to, or want to attend, hearing voices groups. Individual interventions that use the hearing voices approach have been reported as useful, but participants highlighted that these specialised services are difficult to access for many people and poorly understood within mainstream services.^14^ Many voice-hearers have identified a desire to openly discuss the content and meaning of their voices.^15,16^ Given that hearing voices groups and specialised services are unlikely to be available to many voice-hearers, it is important for mental health workers to provide them with the opportunity to hear about and access this evidence-based approach. Nonetheless, mental health workers are often unaware of hearing voices approaches or lack confidence to engage with voice-hearing.^15,17–19^ Voice-hearers have reported experiencing mental health services as disempowering and unhelpful when they provide only ineffective medical options or invalidate voice-hearers’ experiences.^11,16,20^ Researchers have identified a need for materials and resources to support mental health workers in engaging in meaningful conversations with voice-hearers about their voices.^21,22^

The current study evaluates such a resource: Let’s Talk About Voices (LTAV). In this paper we address the research question: What is the impact of engaging with LTAV on workers’ self-reported capacity to work with voice-hearers using a hearing voices approach?

## Method

### Design

A randomised, controlled crossover study design was used. Participants were randomly allocated to one of two groups after completing the first assessment. The immediate group were given access to LTAV immediately, whereas the delayed group received no intervention prior to the second assessment. Both groups were assessed again 4 weeks later, when the delayed group were given access to LTAV. In a further 4 weeks, both groups completed the third and final assessment.

The authors assert that all procedures contributing to this work comply with the ethical standards of the relevant national and institutional committees on human experimentation, and with the Helsinki Declaration of 1975 as revised in 2013. All procedures involving human subjects were approved by The University of Sydney Human Research Ethics Committee (protocol no. 2023-819). The study is reported according to the CONSORT checklist, noting that it is not a clinical trial (as it does not measure a health outcome). Because this was not a clinical trial, prospective registration of the protocol on a publicly accessible platform was not possible. However, all analyses were completed according to the originally approved human research ethics submission.

### Instrument

Because no measure of mental health worker capacity to work with voice-hearers was available, the Supporting Voice-Hearers Measure (SVHM) was developed (Appendix 1). This consists of 23 questions and takes less than 5 min on average to complete. It was co-designed by the researchers, the clinicians who developed LTAV, peer workers and voice-hearers who were familiar with the hearing voices approach. Development was an iterative process that occurred across four workshops. At the first workshop, co-developers brainstormed ideas related to concepts that should be included in the measure. These concepts were then transformed into draft items, which were presented to the co-developers at the second workshop alongside discussion of the Theory of Planned Behaviour (described below). The co-developers revised the item list (removing, adding and changing wording of items). At the third workshop, following presentation of the revised list, co-designers provided further feedback and this was incorporated. Prior to the final workshop, co-designers were provided with a copy of the penultimate version of the measure and piloted completion of the measure on the survey platform. These experiences were discussed at the final workshop, with minor updates suggested and incorporated into the final version.

Questions were developed based on the aims of LTAV and framed around the Theory of Planned Behaviour. The Theory of Planned Behaviour^23–25^ asserts that, assuming a person is able to perform a behaviour, their doing so is predicted by their intention. This intention is determined by the interaction between three factors and the beliefs that shape them. Behavioural beliefs are those about the outcome of a behaviour and how positive and negative that would be, and these determine the person’s attitude toward the behaviour, whether positive or negative. Normative beliefs are those about what other people think about the behaviour and the person’s wish to meet their expectations, resulting in subjective norms or social pressure to engage or not engage in the behaviour. Control beliefs are beliefs about the degree to which internal or external factors will assist or prevent the person from performing the behaviour, leading to perceived behavioural control. While not without its detractors^26^ this theory has been found helpful in explaining, predicting and promoting behaviour change. For example, by 2020 it had been used and examined in more than 4200 papers across a variety of disciplines, including health.^27^ The Theory of Planned Behaviour not only scaffolded instrument development but is also a useful framework through which to interpret the study findings (see Discussion).

Items are rated on a five-point Likert scale, from strongly disagree to strongly agree. Examples of items include: ‘There are many helpful strategies to support voice-hearers’ and ‘The only effective approach to deal with hearing voices is medication’. Negatively worded items are reverse scored so that higher total scores represent more positive responses. Total scores can range from 23 to 115. The measurement properties of the SVHM have been evaluated in a separate study (currently in production) using Rasch analysis. In summary, the SVHM demonstrated suitable measurement properties, including unidimensionality, rating scale validity and overall construct validity. Additionally, the SVHM demonstrated good internal consistency (Cronbach *α* = 0.88), concurrent validity (correlation of 0.49 with overall confidence in working with voice-hearers at baseline) and responsiveness/sensitivity to change.

### LTAV

LTAV was developed in collaboration with voice-hearers and clinical experts, uses a hearing voices approach and is freely available at https://www.nslhd.health.nsw.gov.au/hearingvoices. Mental health workers within the organisation in which it was developed have described it as easy to use and extremely valuable for supporting voice-hearers. It consists of seven short, animated videos and accompanying worksheets covering a range of recovery-focused, strength-based topics. These are voice-hearer facing but designed for flexible use by mental health workers. For example, a worker could refer a voice-hearer to the online resources, use these in a group setting or work through them together with the voice-hearer. They can choose the most relevant resources to use, without needing to utilise them all. Table 1 provides a summary of the topics covered in the videos and worksheets.

**Table 1:** Contents of Let’s Talk About Voices modules^28^

Module no.	Title	Description
1	Introduction to hearing voices	Find out about how common it is to hear voices and explore different voice-hearing experiences
2	The big questions	Explore various explanations for voice-hearing experiences and find answers to some questions
3	Exploring recovery	Explore the concept of recovery and learn about your strengths and values
4	Practical coping strategies	Develop some practical strategies that can be used in coping with distressing voices
5	Navigating the tough times	Learn about triggers, early warning signs and how to pack a toolkit of coping strategies
6	Exploring and explaining voices	Through voice profiling, explore how to reclaim power and control of your life
7	The compassionate approach	Discover how to show yourself and your voices compassion

https://www.nslhd.health.nsw.gov.au/hearingvoices/Pages/Video-series-and-worksheets.aspx.

### Procedure

Upon being given access to LTAV, mental health workers were asked to watch each of the videos and read through all the worksheets within a 7-day period. After 7 days they were sent a reminder that, if they had not done so, to please finish going through LTAV in the next week. They were not required to use the resources with voice-hearers, although they were welcome to do so if they found them helpful.

### Sampling and recruitment

Participants were eligible if they were currently employed as mental health workers (including peer workers), worked with voice-hearers and had no previous experience with LTAV. To avoid contamination, the name of the resource was not revealed until each participant was given access to the resource, at which point they were asked whether they had seen it previously. The few participants who responded in the affirmative were diverted to a related study.

Mental health workers across Australia and other English-speaking countries were recruited through the newsletters and social media of a wide range of professional and mental health organisations. Researchers also sent project flyers to their professional networks with a request to circulate them. Posts were made on social media, such as Facebook and Instagram. People who were interested in participating were invited to follow a link to the participant information sheet and consent form. They indicated their consent using REDCap’s e-consent capability. Participants were randomised to groups via a list generated by an electronic random sample generator. The randomised list was input into the electronic survey tool, which assigned each participant to a group according to the list. Occasionally a participant commenced the first questionnaire twice (usually due to closing prior to completion or losing the link to LTAV following allocation to the immediate group). In these cases, the most complete record was retained and the other deleted; if both records were equally complete, the second was deleted. There was no evidence that this caused bias in group allocation because, in 12 of the 15 cases, both records were allocated to the same group. The intended sample size was 128 full completions, calculated using an estimated effect size of 0.25.

### Data collection

Data were collected via online surveys using REDCap electronic data capture tools hosted at The University of Sydney,^29,30^ between December 2023 and August 2024. The first survey was completed immediately after consent was received, following which participants were informed of their group allocation. Participants received email invitations with links to complete the second and third surveys 4 weeks after completion of each previous survey. They were emailed reminders if they did not complete each survey within 1 week, and were asked to view the resources within 1 week of being given access to LTAV. Demographic and practice data were collected in the first questionnaire.

### Data analysis

The primary analyses were completed using data from participants who had completed measures at the time points included in the relevant analysis. All analyses were completed using IPM SPSS Statistics (version 28.0). Intention-to-treat analyses were also completed to determine whether the preliminary results held true for the full sample. For the intention-to-treat analyses, the most recent observation was carried forward for participants who did not complete all measures (i.e. if a participant had measures only for time 1, these were then carried forward to time 2 and time 3; if a participant had time 1 and time 2 measures, the latter were carried forward to time 3).

### Between-group comparisons

To test for the changes associated with engaging with LTAV resources, an analysis of covariance was conducted comparing time 1 and time 2 data for the immediate and delayed groups. Time 2 scores on the SVHM were included as the dependent variable, with time 1 scores entered as a covariate. The grouping variable was group allocation (i.e. immediate or delayed group). Effect size estimates (partial *η*^2^) were also calculated. Values of 0.01, 0.06 and 0.14 were used as indicators of small, medium and large effect size, respectively.

### Pairwise repeated-measures comparisons

To further explore the influence of LTAV, a series of pairwise comparisons were completed for each group. The immediate and delayed groups were analysed separately, and repeated-measures *t*-tests were completed for time 1 and time 2, time 2 and time 3, and time 1 and time 3. Effect size estimates (Cohen’s *d*) were also calculated. Values of 0.2, 0.5 and 0.8 were used as indicators of small, medium and large effect size, respectively.

### Tests of individual items

To provide greater detail on the changes associated with engaging with the LTAV resources, averages scores for each item before and after access to LTAV (i.e. time 1 and time 2 for the immediate group and time 2 and time 3 for the delayed group) were compared using repeated-measures *t*-tests. Bonferroni correction was used to adjust for the inflated risk of type I errors associated with multiple comparisons. This adjustment (0.05/23) resulted in the significance value being set at *α* = 0.00217 for these analyses. Effect size estimates (Cohen’s *d*) were also calculated.

### Differences in change score according to demographic characteristics

To examine whether access to LTAV had a variable impact on the different participant groups, a series of analyses of variance were also completed. For these analyses, a ‘change score’ was calculated for each participant based on scores before and after receiving access to LTAV (i.e. change from time 1 to time 2 for the immediate group and from time 2 to time 3 for the delayed group). In analysis of variance, change score was the dependent variable and grouping variables were: profession; years working in mental health; work setting; frequency of working with voice-hearers; and confidence in working with voice-hearers at time 1.

## Results

### Participants

**Fig. 1:**
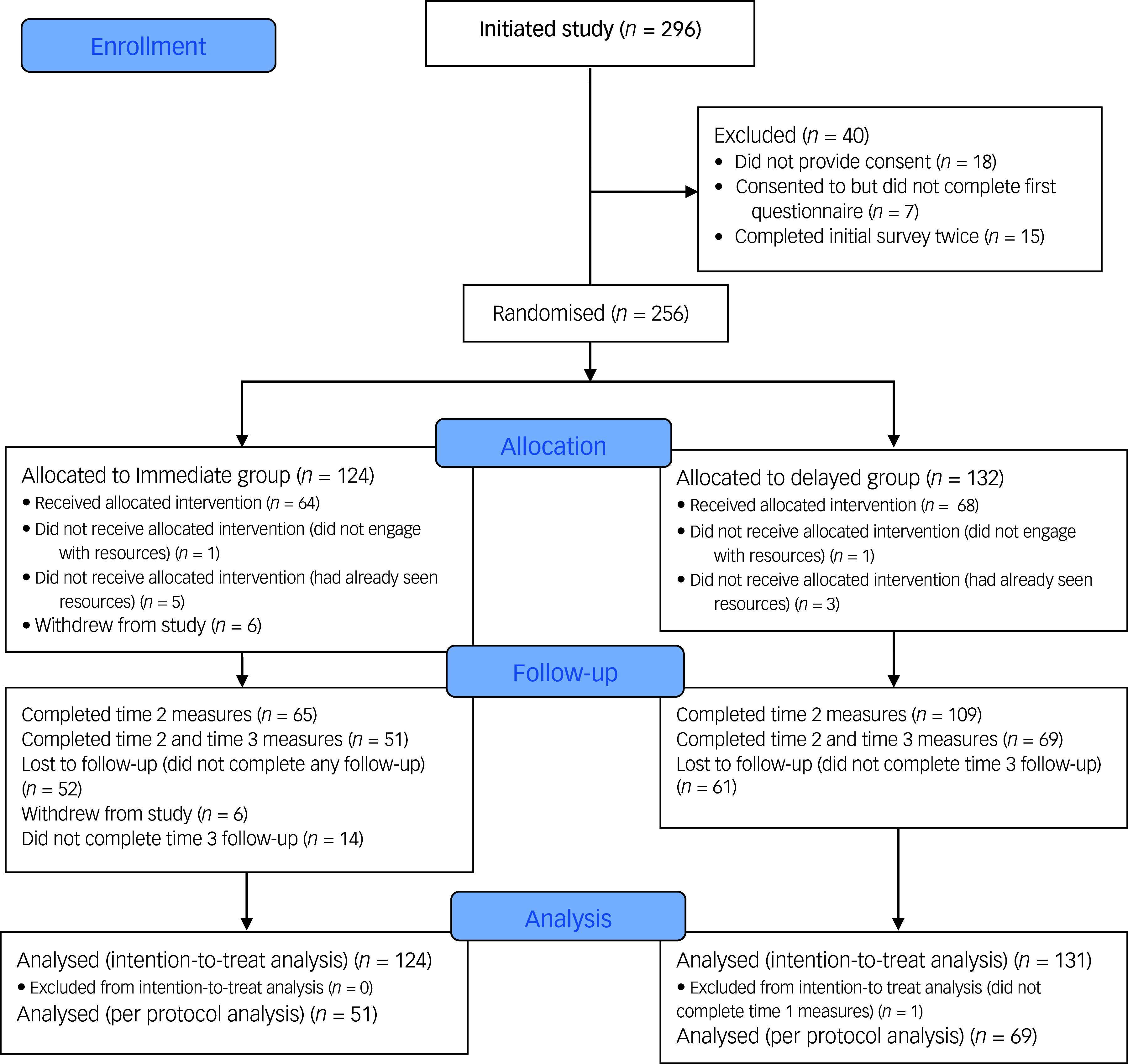
CONSORT flow diagram.

A total of 296 individuals commenced the initial survey. Randomisation and progress through the study are outlined in the CONSORT flow diagram (Fig. 1). Of these, 40 were removed due to either (a) not providing consent to participate, (b) completing the initial survey twice or (c) providing only name and contact email but no other details. This left a total commencing sample of 256 individuals. Of these 256 individuals, 120 provided measures at all 3 time points and an additional 54 provided data at time 1 and time 2 but not at time 3 (of these, 14 were in the immediate group, for whom time 2 represented the post-intervention assessment). As can be seen from the CONSORT diagram, there was significantly higher attrition from the immediate group from time 1 to time 2 compared with the delayed group. This is likely to be related to the former already having received access to the resources, and therefore perhaps having less incentive to complete the time 2 measures. Two participants who completed all measures but did not look at the resources (i.e. were not exposed to LTAV), and eight participants who identified on presentation of the resources that they had previously engaged with the resources were excluded from the primary analyses but were included in the intention-to-treat analyses. Most participants did not provide reasons for withdrawing from the study – they simply failed to respond to the survey invitations. Only six participants, all from the immediate group, cited reasons including insufficient time (*n* = 3), information technology problems, health problems and disagreement with content.

Demographic characteristics of the sample are presented in Table 2. There were no significant differences in the demographic characteristics of completers compared with non-completers. Comparisons of demographics for participants with different patterns of survey completion are presented in Appendix 2.

**Table 2:** Demographic characteristics, all participants (*N* = 256)

Characteristics	Immediate group (*n* = 124) *n* (%)	Delayed group (*n* = 132) *n* (%)
Gender identity		
Man/male	22 (17.7%)	30 (22.7%)
Woman/female	100 (80.6%)	99 (75%)
Transgender, non-binary or does not wish to answer	2 (1.6%)	3 (2.3%)
Profession		
Nurse	13 (10.5%)	11 (8.3%)
Occupational therapist	22 (17.7%)	27 (20.5%)
Peer worker	20 (16.1%)	26 (19.7%)
Psychiatrist	7 (5.6%)	1 (0.7%)
Psychologist	16 (12.9%)	18 (13.6%)
Social worker	9 (7.3%)	20 (15.2%)
Support worker	17 (13.7%)	9 (6.8%)
Manager/administrator	4 (3.2%)	5 (3.8%)
Other^a^	16 (12.9%)	13 (9.8%)
Years working in mental health		
0–1	6 (4.8%)	8 (6.1%)
2–5	46 (37.1%)	57 (43.2%)
6–10	35 (28.2%)	26 (19.7%)
>10	37 (29.8%)	41 (31.1%)
Work setting		
Hospital	26 (21.0%)	23 (17.4%)
Community	90 (72.6%)	89 (67.4%)
Mixed	3 (2.4%)	4 (3.0%)
Other^b^	5 (3.2%)	14 (10.6%)
Frequency of working with voice-hearers		
Very frequently	57 (46%)	48 (36.4%)
Frequently	38 (30.6%)	53 (40.2%)
Sometimes	21 (16.9%)	19 (14.4%)
Occasionally	7 (5.6%)	11 (8.3%)
Never	1 (0.8%)	1 (0.8%)
Confidence in working with voice-hearers		
Not at all confident	2 (1.6%)	4 (3%)
Not very confident	9 (7.3%)	13 (9.8%)
Somewhat confident	57 (46%)	65 (49.2%)
Confident	45 (36.3%)	39 (29.5%)
Very confident	11 (8.9%)	11 (8.3%)
Country of practice		
Australia^c^	102 (82.3%)	103 (78.0%)
USA	7 (5.6%)	16 (12.1%)
UK	3 (2.4%)	4 (3.0%)
Canada	1 (0.8%)	3 (2.3%)
New Zealand	1 (0.8%)	2 (1.5%)
Republic of Ireland	3 (2.4%)	0 (0%)
Singapore	1 (0.8%)	2 (1.5%)
Nigeria	2 (1.6%)	0 (0%)
Other (Eswatini, Ethiopia, Ghana, Hong Kong (SAR), India, Morocco)	4 (3.2%)	2 (1.5%)

a. Other professions include advocate, art therapist, case coordinator, counsellor, recovery coach, dietician, diversional therapist, psychotherapist and student.

b. Other work settings include education, custody, housing, administration and unclear.

c. Australian participants came from New South Wales (*n* = 84), Western Australia (*n* = 29), Victoria (*n* = 28), Queensland (*n* = 21) and South Australia (*n* = 21).

**Fig. 2:**
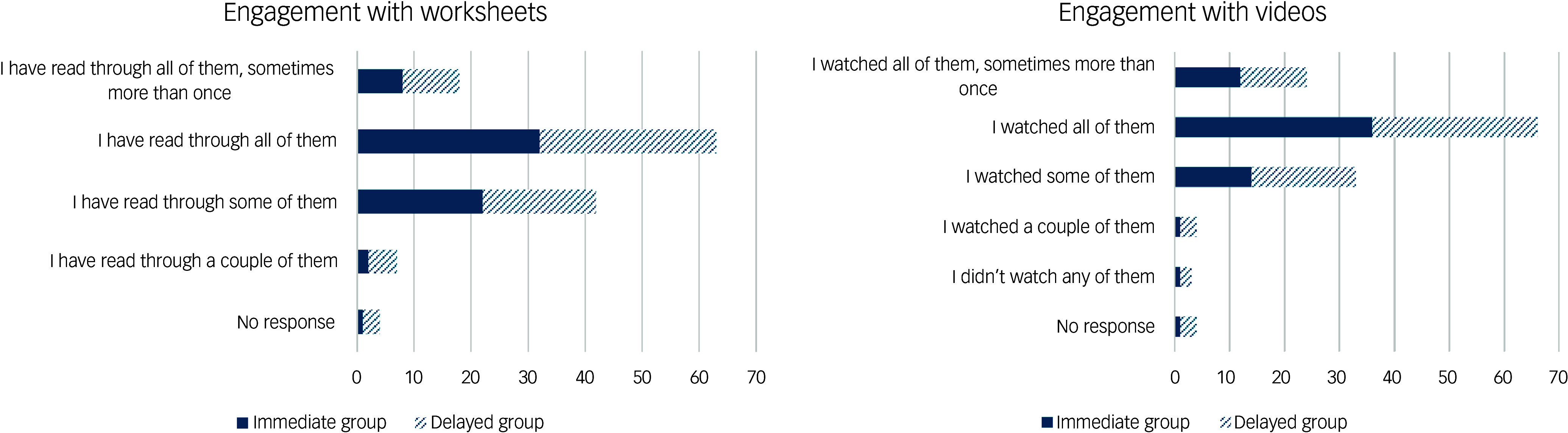
Participant engagement with videos and worksheets.

Figure 2 depicts participants’ reported engagement with the LTAV resources for those included in the primary analysis. While not everyone engaged with all the resources, 67% of participants watched all videos and 60% read all worksheets.

### Between-group comparisons

The between-group comparison, testing the changes associated with engaging with the LTAV resources compared with not having access to them, was highly significant. For the primary analyses, the result was *F*_1,171_ = 40.2, *P* < 0.001, with an effect size (partial *η*^2^) of 0.19 indicating a large effect size. In the intention-to-treat analysis, the result remained significant (*F*_1,252_ = 22.9, *P* < 0.001), with an effect size of 0.08 indicating a medium effect size.

### Pairwise repeated-measures comparisons

**Fig. 3:**
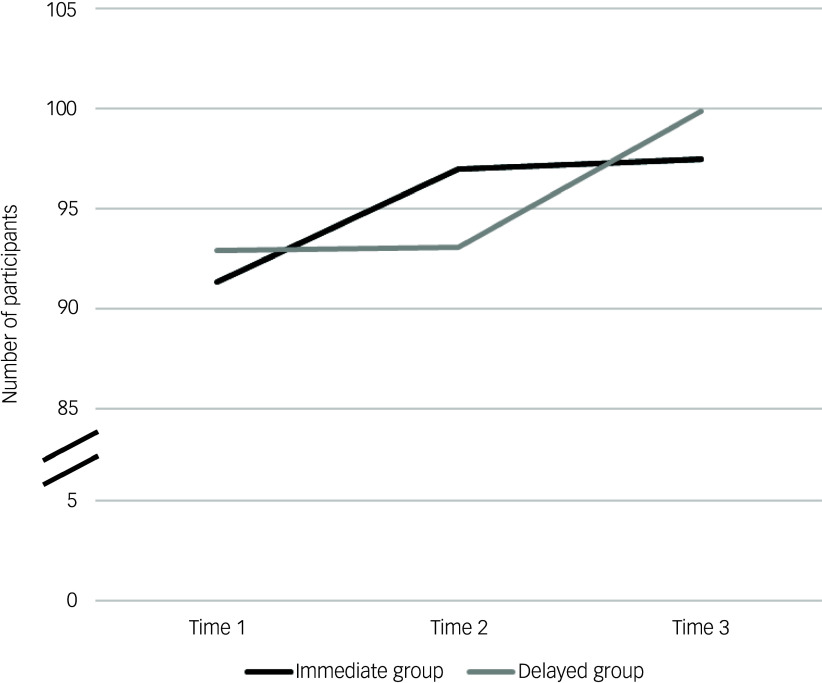
Visual representation of change over time for the Supporting Voice-Hearers Measure, immediate and delayed groups.

Change over time for the immediate and delayed groups is presented in Table 3 and visually summarised in Fig. 3.

**Table 3:** Supporting Voice Hearers Measure scores, across time points for immediate and delayed groups

Time period	Per protocol analysis		Intention-to-treat analysis	
	Immediate group (*n* = 51) *m* (s.d.)	Delayed group (*n* = 69) *m* (s.d.)	Immediate group (*n* = 124) *m* (s.d.)	Delayed group (*n* = 131) *m* (s.d.)
Time 1	91.3 (8.8)	92.9 (10.0)	91.7 (9.0)	92.8 (9.6)
Time 2	97.0 (8.9)	93.1 (10.4)	94.8 (9.8)	92.4 (9.8)^a^
Time 3	97.5 (8.2)	99.9 (8.7)	94.9 (9.7)	95.9 (9.8)^a^

a. For these figures, *n* = 132 because one participant completed the time 2 measure but not time 1 or time 3; this participant was included in intention-to-treat analysis with time 2 data carried over to time 3.

Results from the pair-wise comparisons for each group are summarised in Table 4. For the immediate group, who received access to LTAV between time 1 and time 2, there was significant change from time 1 to time 2 and that change was sustained at time 3. For the delayed group, who received the access to LTAV between time 2 and time 3, there was no change between time 1 and time 2 but a significant change from time 2 to time 3. These results were consistent between the per-protocol and intention-to-treat analyses, with effect sizes (Cohen’s *d*) of approximately 0.9 in the primary analyses (indicating a large effect size) and 0.5 in the intention-to-treat analyses (indicating a medium effect size).

**Table 4:** Results from pre- to post-pairwise comparisons

Group	T1 versus T2		T2 versus T3		T1 versus T3	
	T1, *m* (s.d.)	T2, *m* (s.d.)	T2, *m* (s.d.)	T3, *m* (s.d.)	T1, *m* (s.d.)	T3, *m* (s.d.)
Immediate group^a^	91.7 (8.7)	97.6 (9.5)	97.0 (8.9)	97.5 (8.2)	91.3 (8.8)	97.5 (8.2)
	T1 versus T2 (*n* = 65)		T2 versus T3 (*n* = 51)		T1 versus T3 (*n* = 51)	
	*t* = 7.1, *P* < 0.001, *d* = 0.89		*t* = 0.65, *P* = 0.517		*t* = 6.5, *P* < 0.001, *d* = 0.90	
Delayed group^a^	93.3 (9.7)	92.9 (9.9)	93.1 (10.4)	99.9 (8.7)	92.9 (10.0)	99.9 (8.7)
	T1 versus T2 (*n* = 109)		T2 versus T3 (*n* = 69)		T1 versus T3 (*n* = 69)	
	*t* = −0.7, *P* = 0.468		*t* = 7.3, *P* < 0.001, *d* = 0.87		*t* = 7.6, *P* < 0.001, *d* = 0.92	
Immediate group (ITT)^b^	91.7 (9.0)	94.8 (9.8)	94.8 (9.8)	94.9 (9.7)	91.7 (9.0)	94.9 (9.7)
	T1 versus T2 (*n* = 124)		T2 versus T3 (*n* = 124)		T1 versus T3 (*n* = 124)	
	*t* = 6.1, *P* < 0.001, *d* = 0.55		*t* = 0.7, *P* = 0.257		*t* = 6.3, *P* < 0.001, *d* = 0.57	
Delayed group (ITT)^b^	92.8 (9.6)	92.5 (9.7)	92.4 (9.8)	95.9 (9.8)	92.8 (9.6)	96.0 (9.7)
	T1 versus T2 (*n* = 131)		T2 versus T3 (*n* = 132^c^)		T1 versus T3 (*n* = 131)	
	*t* = −0.7, *P* = 0.249		*t* = 6.1, *P* < 0.001, *d* = 0.54		*t* = 5.0, *P* < 0.001, *d* = 0.44	

T1, time 1; T2, time 2; T3, time 3; ITT, intention-to-treat analysis.

a. Analyses completed including participants who completed measures at both relevant time points.

b. Analyses completed on an intent-to-treat basis (missing time points were replaced with most recent observation).

c. For this analysis, *n* = 132 because one participant completed the T2 measure but not T1 or T3; this participant was included in ITT analysis, with T2 data carried over to T3.

### Tests of individual items

Of the 23 test items, 14 showed significant change between pre- and post-intervention, with 11 having effect size of 0.4 or larger. These are presented in Table 5, grouped by their Theory of Planned Behaviour domain and ordered by effect size.

**Table 5:** Individual items pre- to post-test

Item no.	Item	Pre-test, mean	Post-test, mean	*t*-Statistic	*P*-value	Cohen’s *d*
Behavioural beliefs and attitudes						
12	There are many helpful strategies to support voice-hearers	3.65	4.33	9.1	<0.001*	0.79
22	Exploring voice-hearing experiences can offer enrichment, growth and development	4.23	4.53	5.2	<0.001*	0.46
13	Discussing the meaning of voices is likely to make the problem worse^a^	4.06	4.38	5.0	<0.001*	0.43
8	Hearing about the experiences of other voice-hearers is likely to be beneficial for people who hear voices	4.33	4.60	4.6	<0.001*	0.40
17	It is helpful for voice-hearers to understand their voices as a meaningful experience to be explored rather than just a symptom of illness	4.18	4.45	4.6	<0.001*	0.40
7	People need to block out their voices because they are not real^a^	4.24	4.40	3.0	0.003	0.27
23	To support voice-hearers, it is important that I understand their individual voice-hearing experiences and what the voices mean to them	4.35	4.53	3.0	0.003	0.26
16	The only effective approach to deal with hearing voices is medication^a^	4.34	4.49	2.7	0.008	0.23
1	People can live a good life while continuing to hear voices	4.40	4.51	1.6	0.118	0.14
3	It’s important to be non-judgemental when working with voice-hearers	4.76	4.76	0.0	1.00	0.00
Normative beliefs and subjective norms						
20	Helping people to understand and engage positively with voices is an effective and evidence-based approach	4.04	4.45	6.1	<0.001*	0.53
11	Voice-hearers value being able to discuss their voice-hearing experiences with me	3.71	4.07	5.4	<0.001*	0.47
19	I worry that my professional colleagues would not approve of me encouraging people to explore their voices^a^	3.52	3.66	1.6	0.105	0.14
Control beliefs and perceived behavioural control						
10	I have sufficient knowledge and understanding of voice-hearing to work positively with voice-hearers	3.26	4.01	8.7	<0.001*	0.75
15	I am able to validate people’s experiences of voice-hearing (not necessarily what the voices say)	4.14	4.44	5.0	<0.001*	0.43
2	I feel overwhelmed or out of my depth when people talk to me about their voices^a^	3.84	4.16	4.1	<0.001*	0.35
6	I feel comfortable to raise the topic of voices with the people I work with	4.23	4.43	2.2	0.027	0.19
Behavioural intentions and behaviours						
21	I work with family and friends of voice-hearers to help them to better understand and support the voice-hearer with their voices	3.21	3.62	5.5	<0.001*	0.48
14	I provide information about voice-hearing to help people understand it as a common experience so they will feel less alone	3.83	4.24	5.3	<0.001*	0.46
4	I advise people to ignore their voices or to try to distract themselves^a^	3.50	3.83	4.0	<0.001*	0.34
9	I routinely talk with people about their voices – for example, their personae and what they say	3.54	3.78	3.2	0.002*	0.28
5	I want to help voice-hearers develop a better relationship with their voices	4.41	4.49	1.2	0.245	0.10
18	I want to learn from voice-hearers to help me be a better support	4.65	4.67	0.4	0.663	0.0

a. Negatively worded item, ratings were reversed prior to analysis. *P < 0.00217 (Bonferroni correction applied for 23 comparisons).

Individual items with the lowest significance and effect sizes were those, with one exception, where the initial mean score was relatively high in this sample, indicating limited room for improvement (items 1, 3, 5, 6, 7, 16, 18 and 23).

### Differences in change score according to demographic characteristics

Exploration of change scores across different demographic categories revealed no significant differences according to profession (*F*_8,125_ = 1.69, *P* = 0.108), number of years working in mental health (*F*_3,130_ = 0.89, *P* = 0.450), work setting (*F*_3,130_ = 0.36, *P* = 0.786) or frequency of working with voice-hearers (*F*_4,129_ = 0.67, *P* = 0.616). Comparison of change scores based on ratings of confidence in working with voice-hearers revealed highly significant differences (*F*_4,129_ = 8.0, *P* < 0.001). Average change scores were significantly higher for those who rated themselves as ‘Not at all confident’ (average change score 21.5), ‘Not very confident’ (average change score 13.4) or ’Somewhat confident’ (average change score 6.4) when compared with individuals rating themselves as either ‘Confident’ (average change score 3.8) or ‘Very confident’ (average change score 4.5).

## Discussion

This study is the first, to the authors’ knowledge, to evaluate an online resource that uses a hearing voices approach. It investigated the self-reported confidence and capabilities of mental health workers to work with voice-hearers using this approach. The findings provide an early indication of the potential utility of LTAV, with participants showing significant improvements after accessing LTAV, which were sustained at follow-up, and differences between intervention and control conditions with large effect sizes. Significance remained following intention-to-treat analysis with moderate effect sizes, indicating that the results cannot be attributed to non-random attrition of participants. The apparent impact of LTAV cut across profession, work setting, level of experience and frequency of work with voice-hearers.

While our findings do not guarantee that practitioners will either take a hearing voices approach or use LTAV in their future practice, the individual item analysis provides a positive indication. First, significant changes were observed in the 4 items (items 4, 9, 14 and 21) that reported actual behaviours. Second, even after correcting for family-wise error, significant changes after engaging with LTAV were observed for the majority of items measuring each of the three factors that, according to the Theory of Planned Behaviour, influence behavioural intentions and therefore behaviour.^23–25^ The significantly improved behavioural beliefs and attitudes items may address a particular area of need. For example, mental health workers have expressed fears that talking about voices might make these worse^15,31^ and, in a study of mental health workers’ knowledge and attitudes about hearing voices groups, only 25% were aware of the evidence base for these groups.^17^ A qualitative study of nurses in an acute setting found that distraction was often the tool of choice in dealing with voice-hearing, the impact of which was modest and short term.^21^ Due to volunteer bias, these qualitative studies probably report attitudes of a group of people who are relatively open to hearing voices approaches; beliefs in the benefits of voice-hearing approaches may well be far less widespread among non-volunteers.

Previous studies also indicate the potential impact of improvement in subjective norms. This factor, defined as whether a person thinks that other people who matter to them think that they should use such an approach,^25^ has been found to significantly predict intention to assess distressing voice-hearing.^22^

Lastly, an increase in control beliefs and perceived behavioural control is important given that many mental health workers express lack of confidence in supporting voice-hearers.^17,19,21,31–33^ It is encouraging that people who expressed less confidence with working with voice-hearers on intake showed particularly strong improvement in our study, compared with more confident participants. Previous research has shown that providing useful voice-hearing information to voice-hearers is an area in which clinicians across services have indicated relatively low self-efficacy.^22^

That items measuring attitudes, subjective norms and perceived behavioural control (or self-efficacy) all showed significant improvement following engagement with LTAV bodes well for the future practice of participating mental health workers. Even if they do not choose to use LTAV in the future, the change in their attitudes towards, and confidence in working with, voice-hearers may well be beneficial for the latter.

It is clear from the implementation science literature, however, that more than individual change is required if hearing voices approaches are to be adopted into routine practice: organisational and policy commitment is also needed. A systematic review of studies of new implementation of recovery-based practice into adult mental health services found that implementation was affected by setting factors such as the culture, structural characteristics and leadership commitment within the organisation.^34^ Similarly, mental health workers have talked about how the expectations and practices of the organisation and more senior staff influence how they work with voice-hearers.^19^ Perhaps a review of these resources by senior colleagues would support greater organisational commitment. Future research examining workplace cultures and the perspectives of mental health workers about barriers to such an approach in the workplace would provide helpful information in assisting with implementation.

### Limitations

As with any research relying on volunteers, people who agreed to participate are likely to at least have an interest in non-pharmacological approaches to voice-hearing. However, given that those who did not are unlikely to engage with LTAV in a real-world context, the results are likely to reflect the impact on the target market for the resources. Additionally, the studies use of a self-report measure of confidence and capabilities, rather than an objective measure of skills, is another limitation, because self-ratings may not always translate into improved practice. Finally, increasing the self-reported confidence and capabilities of mental health workers in using a hearing voices approach, and providing them with the resources to do so, does not automatically translate into positive outcomes for voice-hearers. Our research team is engaged in ongoing research to assess the impact on voice-hearers of engaging with LTAV.

Notwithstanding the limitations, this study provides preliminary evidence of the usefulness of LTAV, a freely accessible set of videos and accompanying worksheets that mental health workers can use with voice-hearers. The findings suggest that accessing and reviewing these resources results in positive changes, not only to mental health workers’ self-reported behaviour with regard to supporting voice-hearers, but also to their attitudes towards and confidence in doing so.

## Supporting information

Honey et al. supplementary material 1 Honey et al. supplementary material

Honey et al. supplementary material 2 Honey et al. supplementary material

## Data Availability

Anonymised data to support the findings are available upon reasonable request from the corresponding author (A.H.).

## Supplementary material

The supplementary material can be found online at https://doi.org/10.1192/bjo.2025.10071
