# Supplementary material for: Let’s Talk About Voices: randomised controlled crossover study of a resource to support mental health workers in supporting voice-hearers
Source: BJPsych Open. 2025 Jul 7;11(4):e139. doi: 10.1192/bjo.2025.10071 (PMC12247076; doi:10.1192/bjo.2025.10071)
Supplement: Honey et al. supplementary material 1 — Honey et al. supplementary material [file S2056472425100719sup001.docx]

# Appendix 1: Supporting Voice Hearers Measure

**Instruction**: Please rate your level of agreement with the following statements.

**Response options**: Strongly disagree, Disagree, Neither agree nor disagree, Agree, Strongly agree.

**Statements**:

| Item # | Statement | TPB item |
| --- | --- | --- |
| 1 | People can live a good life while continuing to hear voices | Behavioural beliefs |
| 2 | I feel overwhelmed or out of my depth when people talk to me about their voices | Control beliefs |
| 3 | It's important to be non-judgmental when working with voice hearers | Attitudes |
| 4 | I advise people to ignore their voices or to try to distract themselves | Behaviour |
| 5 | I want to help voice-hearers to develop a better relationship with their voices | Behavioural intention |
| 6 | I feel comfortable to raise the topic of voices with the people I work with | Perceived behavioural control |
| 7 | People need to block out their voices because they are not real | Behavioural beliefs |
| 8 | Hearing about the experiences of other voice-hearers is likely to be beneficial for people who hear voices | Behavioural beliefs |
| 9 | I routinely talk with people about their voices, for example, their personas and what they say | Behaviour |
| 10 | I have enough knowledge and understanding of voice hearing to work positively with voice hearers | Perceived behavioural control |
| 11 | Voice hearers value being able to discuss their voice hearing experiences with me | Normative beliefs |
| 12 | There are many helpful strategies to support voice-hearers | Behavioural beliefs |
| 13 | Discussing the meaning of voices is likely to make the problem worse | Behavioural beliefs |
| 14 | I provide information about voice hearing to help people understand it as a common experience so they will feel less alone | Behaviour |
| 15 | I am able to validate people's experiences of voice hearing (not necessarily what the voices say) | Perceived behavioural control |
| 16 | The only effective approach to deal with hearing voices is medication | Behavioural beliefs |
| 17 | It is helpful for voice hearers to understand their voices as a meaningful experience to be explored rather than just a symptom of illness | Attitudes |
| 18 | I want to learn from voice-hearers to help me be a better support | Behavioural intentions |
| 19 | I worry that my professional colleagues would not approve of me encouraging people to explore their voices | Subjective norms |
| 20 | Helping people to understand and engage positively with voices is an effective and evidence based approach | Normative beliefs |
| 21 | I work with family and friends of voice-hearers to help them to better understand and support the voice hearer with their voices | Behaviour |
| 22 | Exploring voice hearing experiences can offer enrichment, growth and development | Behavioural beliefs |
| 23 | To support voice hearers it is important that I understand their individual voice-hearing experiences and what they the voices mean to them | Behavioural beliefs |
