# Supplementary material for: Let’s Talk About Voices: randomised controlled crossover study of a resource to support mental health workers in supporting voice-hearers
Source: BJPsych Open. 2025 Jul 7;11(4):e139. doi: 10.1192/bjo.2025.10071 (PMC12247076; doi:10.1192/bjo.2025.10071)
Supplement: Honey et al. supplementary material 2 — Honey et al. supplementary material [file S2056472425100719sup002.docx]

# Appendix 2: Demographic characteristics by completion status

|  | **All participants (N = 256)** | | **T1 & T2 (N = 174)** | | **T1, T2, T3 (N = 120)** | |
| --- | --- | --- | --- | --- | --- | --- |
|  | Group A  (n = 124) n (%) | Group B  (n = 132)  n (%) | Group A  (n = 65)  n (%) | Group B  (n = 109)  n (%) | Group A  (n = 51)  n (%) | Group B  (n = 69)  n (%) |
| ***Gender identity*** |  |  |  |  |  |  |
| Man/ male | 22 (17.7%) | 30 (22.7%) | 10 (15.4%) | 22 (20.2%) | 6 (11.8%) | 16 (23.2%) |
| Woman/ Female | 100 (80.6%) | 99 (75%) | 55 (84.6%) | 85 (78%) | 45 (88.2%) | 52 (75.4%) |
| Transgender | 1 (0.8%) | 0 | 0 | 0 | 0 | 0 |
| Non-binary | 0 | 3 (2.3%) | 0 | 2 (1.8%) | 0 | 1 (1.4%) |
| I do not wish to answer | 1 (0.8%) | 0 | 0 | 0 | 0 | 0 |
| ***Profession*** |  |  |  |  |  |  |
| Nurse | 13 (10.5%) | 10 (7.6%) | 7 (10.8%) | 9 (8.3%) | 6 (11.8%) | 5 (7.2%) |
| Occupational Therapist | 22 (17.7%) | 27 (20.5%) | 14 (21.5%) | 24 (22%) | 12 (23.5%) | 15 (21.7%) |
| Peer worker | 20 (16.1%) | 26 (19.7%) | 9 (13.8%) | 23 (21.1%) | 7 (13.7%) | 17 (24.6%) |
| Psychiatrist | 5 (4%) | 0 | 3 (4.6%) | 0 | 3 (5.9%) | 0 |
| Psychologist | 16 (12.9%) | 18 (13.6%) | 10 (15.4%) | 15 (13.8%) | 7 (13.7%) | 9 (13%) |
| Social worker | 9 (7.3%) | 20 (15.2%) | 4 (6.2%) | 16 (14.7%) | 3 (5.9%) | 10 (14.5%) |
| Support worker | 17 (13.7%) | 8 (6.1%) | 8 (12.3%) | 4 (3.7%) | 6 (11.8%) | 3 (4.3%) |
| Other | 22 (17.7%) | 23 (17.4%) | 10 (15.4%) | 18 (16.5%) | 7 (13.7%) | 10 (14.5%) |
| ***Years working in mental health*** | | | | | | |
| 0-1 year | 6 (4.8%) | 8 (6.1%) | 2 (3.1%) | 5 (4.6%) | 0 | 4 (5.8%) |
| 2-5 years | 46 (37.1%) | 57 (43.2%) | 26 (40%) | 47 (43.1%) | 20 (39.2%) | 26 (37.7%) |
| 6-10 years | 35 (28.2%) | 26 (19.7%) | 15 (23.1%) | 21 (19.3%) | 13 (25.5%) | 12 (17.4%) |
| More than 10 years | 37 (29.8%) | 41 (31.1%) | 22 (33.8%) | 36 (33%) | 18 (35.3%) | 27 (39.1%) |
| ***Work setting*** |  |  |  |  |  |  |
| Inpatient acute | 19 (15.3%) | 14 (10.6%) | 10 (15.4%) | 10 (9.2%) | 9 (17.6%) | 3 (4.3%) |
| Inpatient rehabilitation | 7 (5.6%) | 5 (3.8%) | 3 (4.6%) | 4 (3.7%) | 2 (3.9%) | 2 (2.9%) |
| Community | 85 (68.5%) | 80 (60.6%) | 45 (69.2%) | 70 (64.2%) | 35 (68.6%) | 49 (71%) |
| Other | 13 (10.5%) | 33 (25%) | 7 (10.8%) | 25 (22.9%) | 5 (9.8%) | 15 (21.7%) |
| ***Frequency of working with voice hearers*** | | | | | | |
| Very frequently | 57 (46%) | 48 (36.4%) | 27 (41.5%) | 40 (36.7%) | 24 (47.1%) | 23 (33.3%) |
| Frequently | 38 (30.6%) | 53 (40.2%) | 21 (32.3%) | 44 (40.4%) | 18 (35.3%) | 29 (42%) |
| Sometimes | 21 (16.9%) | 19 (14.4%) | 12 (18.5%) | 16 (14.7%) | 8 (15.7%) | 10 (14.5%) |
| Occasionally | 7 (5.6%) | 11 (8.3%) | 5 (7.7%) | 8 (7.3%) | 1 (2%) | 6 (8.7%) |
| Never | 1 (0.8%) | 1 (0.8%) | 0 | 1 (0.9%) | 0 | 1 (1.4%) |
| ***Confidence in working with voice hearers*** | | | | | | |
| Not at all confident | 2 (1.6%) | 4 (3%) | 1 (1.5%) | 1 (0.9%) | 1 (2%) | 1 (1.4%) |
| Not very confident | 9 (7.3%) | 13 (9.8%) | 5 (7.7%) | 10 (9.2%) | 4 (7.8%) | 8 (11.6%) |
| Somewhat confident | 57 (46%) | 65 (49.2%) | 35 (53.8%) | 55 (50.5%) | 27 (52.9%) | 35 (50.7%) |
| Confident | 45 (36.3%) | 39 (29.5%) | 22 (33.8%) | 33 (30.3%) | 17 (33.3%) | 19 (27.5%) |
| Very confident | 11 (8.9%) | 11 (8.3%) | 2 (3.1%) | 10 (9.2%) | 2 (3.9%) | 6 (8.7%) |
